# Contrasting behavior of heterochromatic and euchromatic chromosome portions and pericentric genome separation in pre-bouquet spermatocytes of hybrid mice

**DOI:** 10.1007/s00412-014-0479-4

**Published:** 2014-08-15

**Authors:** Harry Scherthan, Karina Schöfisch, Thomas Dell, Doris Illner

**Affiliations:** 1 Institut für Radiobiologie der Bundeswehr in Verb. mit der Univ. Ulm, 80937 München, Germany; 2 Max-Planck-Institut für Molekulare Genetik, Ihnestr. 73, 14195 Berlin, Germany; 3 FB Biologie der TU Kaiserslautern, 67663 Kaiserslautern, Germany; 4 Present Address: 66121 Saarbrücken, Germany; 5 Present Address: Max-Planck-Institut für Stoffwechselforschung, 50931 Köln, Germany

## Abstract

The spatial distribution of parental genomes has attracted much interest because intranuclear chromosome distribution can modulate the transcriptome of cells and influence the efficacy of meiotic homologue pairing. Pairing of parental chromosomes is imperative to sexual reproduction as it translates into homologue segregation and genome haploidization to counteract the genome doubling at fertilization. Differential FISH tagging of parental pericentromeric genome portions and specific painting of euchromatic chromosome arms in *Mus musculus* (MMU) × *Mus spretus* (MSP) hybrid spermatogenesis disclosed a phase of homotypic non-homologous pericentromere clustering that led to parental pericentric genome separation from the pre-leptoteneup to zygotene stages. Preferential clustering of MMU pericentromeres correlated with particular enrichment of epigenetic marks (H3K9me3), HP1-γ and structural maintenance of chromosomes SMC6 complex proteins at the MMU major satellite DNA repeats. In contrast to the separation of heterochromatic pericentric genome portions, the euchromatic arms of homeologous chromosomes showed considerable presynaptic pairing already during leptotene stage of all mice investigated. Pericentric genome separation was eventually disbanded by telomere clustering that concentrated both parental pericentric genome portions in a limited nuclear sector of the bouquet nucleus. Our data disclose the differential behavior of pericentromeric heterochromatin and the euchromatic portions of the parental genomes during homologue search. Homotypic pericentromere clustering early in prophase I may contribute to the exclusion of large repetitive DNA domains from homology search, while the telomere bouquet congregates and registers spatially separated portions of the genome to fuel synapsis initiation and high levels of homologue pairing, thus contributing to the fidelity of meiosis and reproduction.

**Electronic supplementary material:**

The online version of this article (doi:10.1007/s00412-014-0479-4) contains supplementary material, which is available to authorized users.

## Introduction

Pairing of homologous chromosomes during meiosis is essential to fertility as it allows parental chromosomes to segregate and to generate haploid gametes or spores. During the onset of prophase I, intranuclear chromosome architecture is profoundly changed, which involves the attachment of telomeres to the nuclear envelope and chromosome extension and axis formation (Rasmussen and Holm 1980; Scherthan 2007). The spatial distribution of parental chromosome sets and homologous chromosome pairs is transformed into tight synaptic pairing during the zygotene stage of the first meiotic prophase. Recent reports suggest that cohesin and/or Spo11 contribute to considerable prealignment of homologues in pre-zygotene nuclei in mouse spermatogenesis (Boateng et al. 2013; Ishiguro et al. 2014). In yeast and plants, on the other hand, there is early meiotic homology-independent coupling of centromeres which requires the meiotic ZIP1 SC protein and REC8 cohesin in yeast (Bardhan et al. 2010; Tsubouchi and Roeder 2005), and in maize, an SMC6 structural maintenance of chromosomes (SMC) protein homologue (Zhang et al. 2013). Centromere coupling seems to install a premeiotic nuclear architecture that sets an early stage for the meiotic pairing dance (Moore and Shaw 2009; Richards et al. 2012). These observations imply a differential behavior of centromeric/pericentromeric and euchromatic genome portions during the onset of first meiotic prophase. Both the association of non-homologous centromeres and the formation of the telomere bouquet seem to direct the complex choreography of the homologue pairing process (Klutstein and Cooper 2014; Obeso et al. 2014; Scherthan 2001).

In contrast to yeast and plants, premeiotic cells (spermatogonia) and early prophase I spermatocytes of the mouse fail to display an overt non-homologous coupling of centromeres (kinetochores; Bisig et al. 2012). However, there is a significant higher order clustering of pericentromeric heterochromatin (PCH) regions leading to the formation of distinct PCH clusters (chromocenters) well below the diploid chromosome/centromere number (2*n* = 40) of mitotic and meiotic mouse cells (Brero et al. 2005; Hsu et al. 1971; Scherthan et al. 1996). All mouse chromosomes are acrocentric, and pericentromeric regions are abutted with a telomere (Kipling et al. 1991). During the mouse preleptotene stage, telomeres attach to the nuclear envelope (NE) and move to cluster during the zygotene transition (Scherthan et al. 1996). During this process, there is a considerable portion of cells that display presynaptic pairing of homologous euchromatic chromosome arms (Boateng et al. 2013; Ishiguro et al. 2014). Since human spermatocytes do not show a prealignment/pairingof leptotene homologues (Scherthan et al. 1998), it is conceivable that in the mouse a bundling of proximal chromosome ends into large chromocenters by PCH clustering may contribute to this prealignment of homologs prior to synaptic pairing (Ishiguro et al. 2014).

Chromocenter formation in mouse cells depends on epigenetic modifications specific to heterochromatin, like histone methyltransferase-dependent histone H3K9 tri-and di-methylation (Peters et al. 2001; Tachibana et al. 2007), heterochromatin-associated proteins like MeCP2 (Brero et al. 2005) and HP1 family proteins which promote methyl-K9-H3-dependent silencing (see Jenuwein and Allis 2001; Lomberk et al. 2006). In agreement, the disruption of histone methyltransferases or HP1-gamma leads to aberrant chromocenter formation, synapsis defects and infertility in mice (Peters et al. 2001; Tachibana et al. 2007; Takada et al. 2011).

The pericentromeric region of *Mus musculus* (MMU) chromosomes contains megabases of major satellite (sat) DNA repeats (Vissel and Choo 1989), which is functionally separate from the active centromere (kinetochore) domain that is formed by a relatively small amount of minor sat DNA repeats (Guenatri et al. 2004). *Mus spretus* (MSP) pericentromeres, on the other hand, contain large amounts of minor satellite DNA (Wong et al. 1990) and a small amount of major sat DNA (Narayanswami et al. 1992). Differential minor and major sat DNA FISH can track the distribution of both satellite DNAs with the minute MMU minor sat signals being surrounded by vast amount of major sat DNA in MMU nuclei (Guenatri et al. 2004). Investigation of the large pericentric minor and major satellite FISH signals in hybrid spermatogenesis has revealed homeologous pericentromere pairing at pachytene and homotypic clustering of parental pericentric genomes in haploid nuclei of MMU × MSP F1 spermatids (Mayer et al. 2000).

Here, we investigated the repositioning of parental pericentric chromosome sets in premeiotic and early meiotic prophase spermatocytes of *Mus musculus*, *M. spretus* and their interspecific F1 hybrid. In MMU × MSP F1 hybrid spermatogenesis prophase I progresses to metaphase I, with a large fraction of MI cells undergoing cell death owing to disturbed recombination and XY synapsis (Matsuda et al. 1992; Oka et al. 2010). Still, there is a small fraction of spermatocytes that passes the Meiosis I and II divisions and forms dysfunctional haploid spermatids (Matsuda et al. 1991; Oka et al. 2010; Mayer et al. 2000).

Data on the relative repositioning of parental genomes during the onset of prophase I and the bouquet stage of mammals are absent but could shed new light on the homologue pairing process in mammalian meiosis. We, therefore, tagged pericentric haploid parental genome sets during prophase I of MMU × MSP F1 spermatogenesis and observed a phase of separation of parental pericentric heterochromatin regions during the preleptotene and leptotene substages, contrasting with a substantial amount of presynaptic pairing of the euchromatic portion of homeologous (homologous in the parents) leptotene chromosomes. Pericentric genome separation was eventually transformed into homeologous pairing during the bouquet stage of hybrid meiosis.

## Results

### Homeologous pairing initiates during the leptotene/zygotene transition

In mouse meiosis, pericentromeric heterochromatin (PCH) regions have been observed to undergo a dynamic higher order clustering leading to the formation of a few large chromocenters during leptotene and zygotene stages (Scherthan et al. 1996; Takada et al. 2011), with the number of PCH clusters being well below the diploid number of mouse centromeres (2*n* = 40), suggesting the unspecific bundling of proximal chromosome ends prior to synapsis initiation. Differential FISH-tagging of major satellite (sat) and minor sat DNAs at MMU and MSP pericentromeres, respectively, allows the highlighting of the parental pericentric genomes in MMU × MSP F1 nuclei (Mayer et al. 2000). In our experiments, major sat DNA FISH formed large signals at the pericentromeres of MMU metaphase chromosomes, while minor sat FISH showed strong labelling of MSP pericentromeres (Fig. S1A). MMU chromosomes also possess minor sat sequences below the kinetochore domain (Narayanswami et al. 1992; Wong et al. 1990), but these regions were only faintly, if at all, stained in the large major sat PCH domain with our FISH protocol (Fig. S1) that used only mild denaturation and a short renaturation period (“Materials and methods” section).

**Fig. 1:**
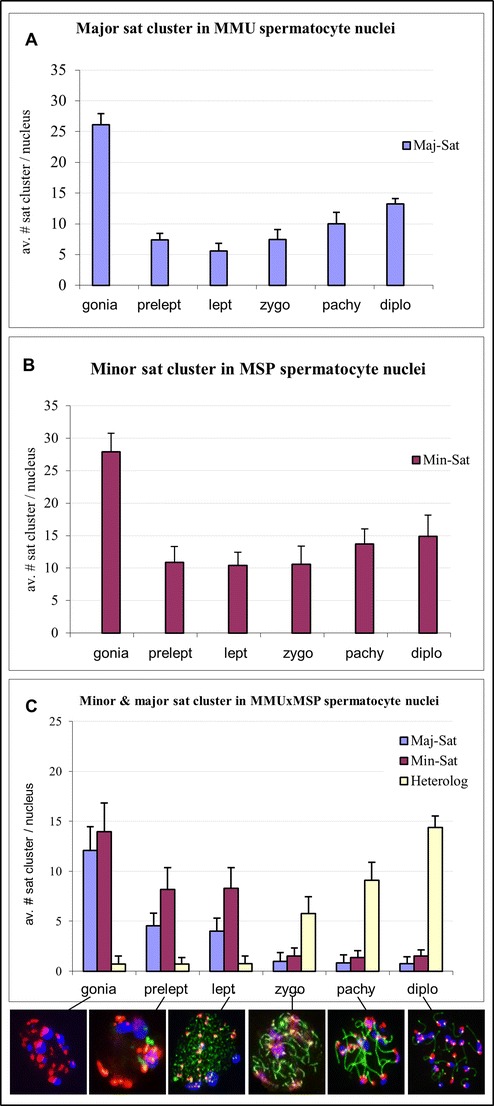
Number of FISH signal clusters of minor and major satellite DNA during meiotic prophase in nuclei from testis suspensions. **a** There are in average 26 major sat clusters in MMU spermatogonia (gonia). This number is reduced in prophase I substages, especially in leptotene when large chromocenters form. **b** Average minor sat cluster number distribution in MSP testis cells reveals a pattern similar to MMU, but with somewhat elevated signal numbers/nucleus. **c** Average number of minor (*red*), major (*blue*), and heterologous clusters (*yellow*) during MMU × MSP F1 spermatogenesis showing low heterologous pairing in gonia nuclei up to leptotene. From zygotene onwards, the amount of heterologous FISH signal clusters increases significantly, reflecting the course of homeologous pairing. The *image row below* **c** shows the staging of the nuclei by minor (*red*, Cy3) and major (*blue*, Cy5) sat FISH in combination with SYCP3 IF staining (*green*). Cells with small clusters of SYCP3 were addressed as preleptotene

In testis suspension cells, costained for the SYCP3 axial element protein (Lammers et al. 1994), minor and major sat FISH readily revealed parental pericentric genome positioning prior to and during prophase I (Fig. 1). In premeiotic cells (spermatogonia), we observed in average 26 randomly distributed major sat signals in MMU and 28 minor sat signals in MSP spermatogonia (*n* ≥ 20 cells; Fig. 1a, b), indicating that most pericentromeres are not associated with each other prior to the onset of meiosis, agreeing with previous reports (Bisig et al. 2012; Scherthan et al. 1996). During the preleptotene and leptotene stages, there were in average 7 and 6 large major sat FISH signal clusters/cell in MMU spermatocytes, respectively, while 11 large minor sat FISH clusters per pre/leptotene nucleus were noted in MSP spermatocytes (Fig. 1a, b). These data indicate that in both parental mouse species 4 or more meiotic pericentromeres associate in one PCH cluster.

In MMU zygotene and pachytene spermatocytes there were in average 7 and 10 satellite signals/nucleus, respectively (Fig. 1a). In contrast, MSP spermatocytes displayed in average 10 and 14 minor sat clusters per zygotene and pachytene nucleus (Fig. 1b), respectively. These observations reveal that the MMU major satellite, which forms megabases of AT-rich tandem arrays (Vissel and Choo 1989), tends to undergo an intensive clustering leading to an average of 5 large aggregates (chromocenters) in MMU leptotene and zygotene spermatocyte nuclei (Fig. 1a). In contrast, clustering is less prominent for MSP minor sat pericentromeres that form in average 10 clusters/zygotene spermatocyte (Fig. 1b). These data suggest that pericentric associations in both mouse species can involve ≥4 chromosomes per PCH cluster, being reminiscent of observations in MMU mitotic interphase cells (Guenatri et al. 2004).

MMU × MSP F1 hybrid spermatogonia displayed in average 12 major and 14 minor sat FISH clusters/nucleus, while there usually was only 1 heterologous cluster (*n* ≥ 20 cells; Fig. 1c). This distribution pattern suggests that in F1 hybrid spermatogenesis, there are parent-specific non-homologous pericentromeric genome associations prior to and at the onset of meiotic prophase up to the end of the leptotene stage (Fig. 1c). At the onset of zygotene stage, there were in average 6 differentially colored pericentric signals, while SCP3 costaining revealed the commencement of synaptic pairing of homeologous parental chromosomes (Fig. 1c). F1 pachytene nuclei displayed SYCP3-positive SCs indicating tight synaptic pairing of homeologous parental chromosomes, while still there were in average 14 PCH clusters/pachytene nucleus, indicating persistance of higher order PCH clustering in late murine prophase I (Fig. 1c).

### Telomere clustering induces intense homeologous chromatin contacts in the bouquet stage

**Fig. 2:**
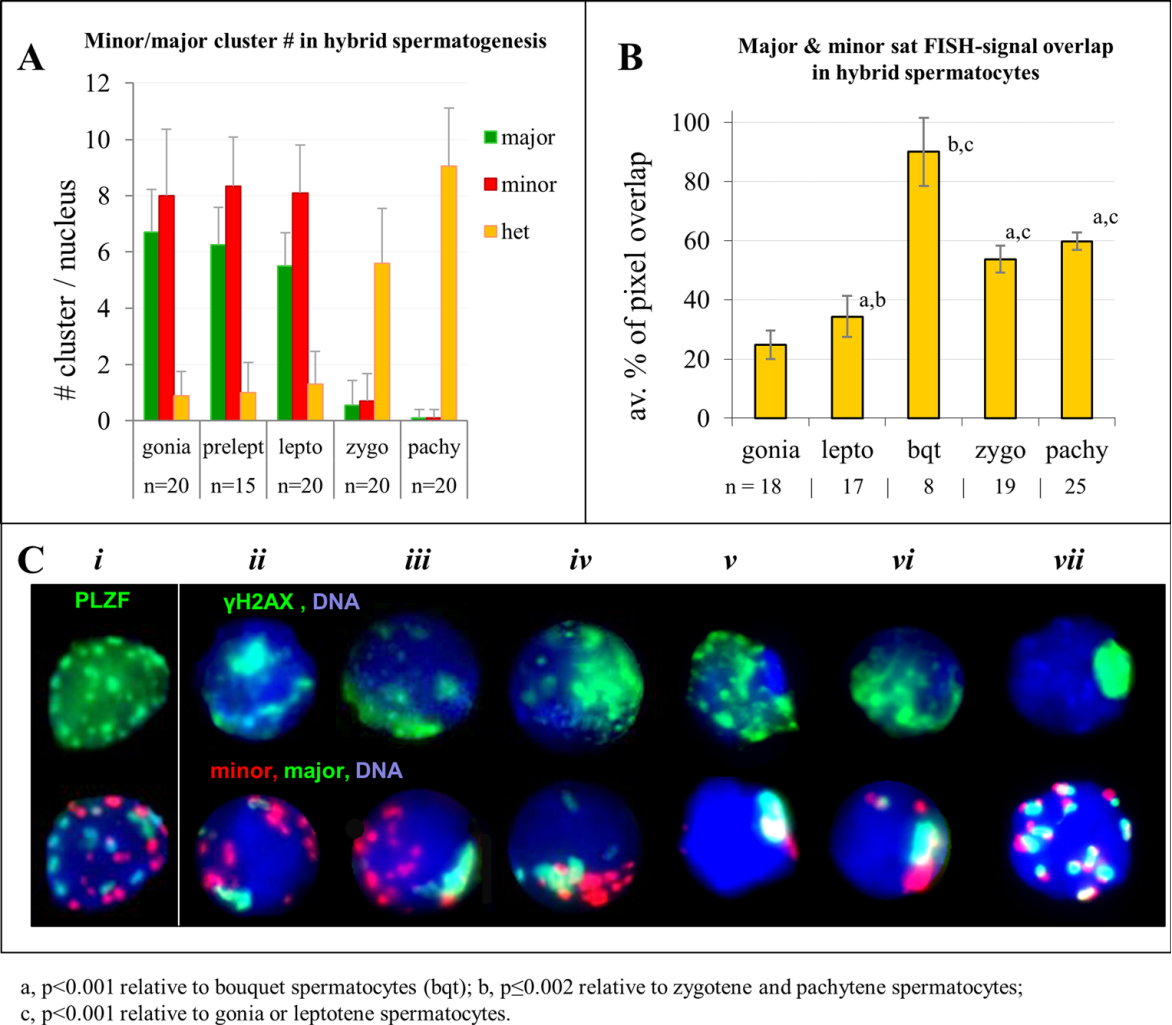
Investigation of parental pericentromere genome distribution by sat DNA FISH and stage-specific γH2AX staining (Mahadevaiah et al. 2001) in hybrid spermatogenesis. **a** Frequency of minor (*red*) and major (*green*) FISH signal clusters in prophase I substages from 10 dpp MMU × MSP F1 testis sections. Heterologous signal pairs (*orange columns*) are increased in zygotene and pachytene nuclei. **b** Percentage of FISH signal overlap as determined by pixel colocalization obtained from 3D reconstructions of cLSM images using the Nemo imaging program (Iannuccelli et al. 2010; c.f. Fig. 4) and expressed as percentage of total pixel overlap per nucleus. Consistent with the absence of heterologous pericentromere pairing, spermatogonia (gonia) and leptotene nuclei display only a low amount of signal overlap (MMU pericentromeres also contain a small amount of centromeric minor sat DNA). Tight bouquet stage cells (**c**,*v*) display ∼90 % of minor/major signal overlap, indicating intensive chromatin intermingling of the proximal chromosome ends in the limited nuclear space of the bouquet base. Zygotene and pachytene nuclei show less signal overlap due to relaxed chromatin organization after tight telomere clustering (see Scherthan et al. 1998). **c** Detailed analysis of parental genome pairing during leptotene–zygotene. In PLZF-tagged (*green*) spermatogonia, *i* pericentric parental genomes (MMU major sat, *green*; MSP minor sat, *red*) are largely without heterologous contacts. **c** *ii–vi* Zygotene nuclei, as identified by patchy γH2AX fluorescence (*upper row*), display a growing degree of pericentric FISH signal clustering during tight bouquet formation (for telomere staging, see Fig. 3). Major sat signals of heterochromatin clusters are usually void of γH2AX signal (e.g., *v*). **c** *vii* Pachytene nucleus show numerous heterologously colored FISH signal pairs indicating homeologous pairing

**Fig. 3:**
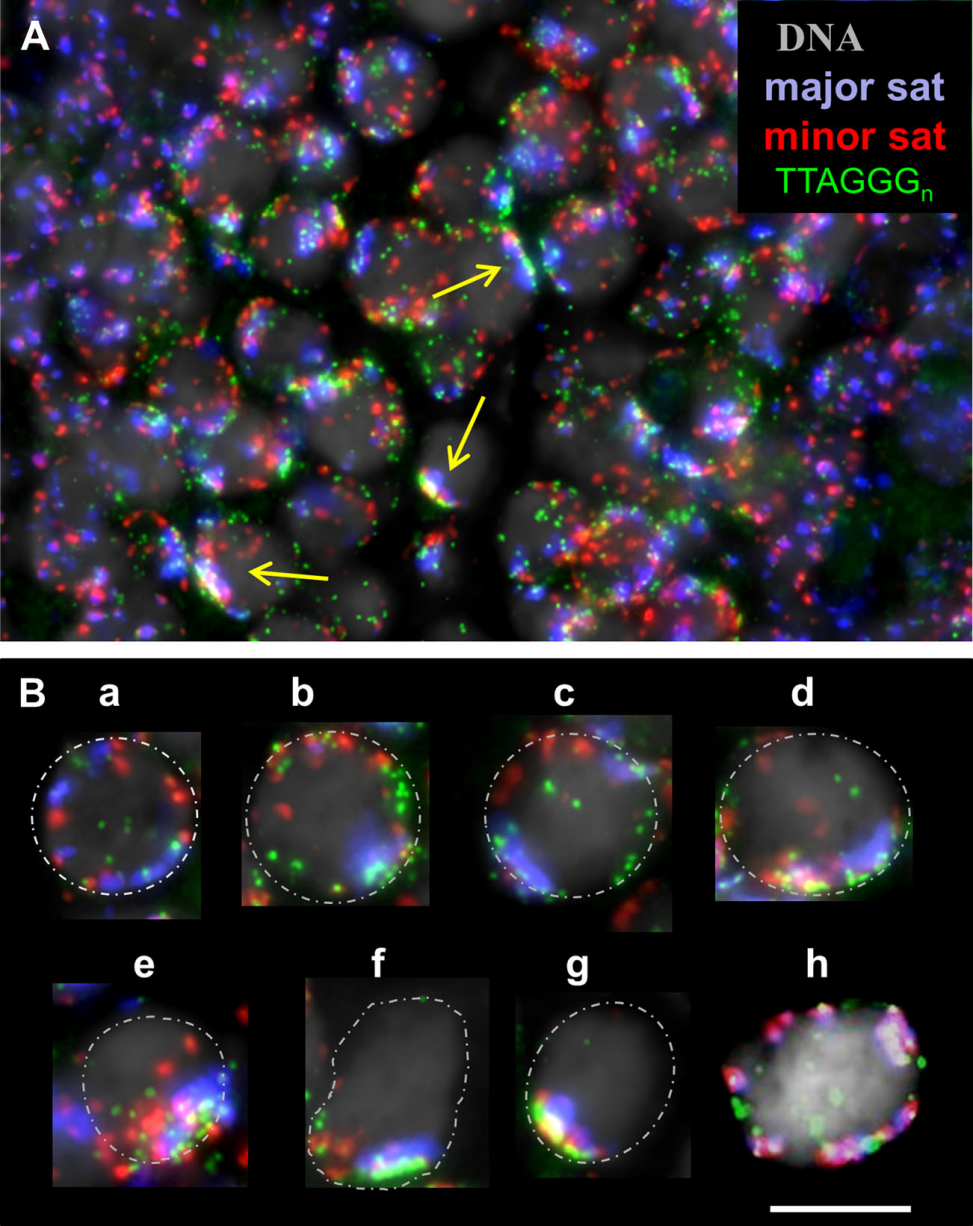
**a** Triple-colorFISH of a paraffin section of a 10-dpphybrid testis revealing major sat (*blue*), minor sat (*red*), and TTAGGG telomeres (*green*) in bouquet nuclei (*arrowed*; DAPI displayed (*gray*). Nuclei with tightly clustered major and minor sat signals are in tight bouquet stage (*arrows*). **b** Telomere and parental pericentromere clustering during homology search in MMU × MSP F1 spermatocytes selected from paraffin sections. Nuclei of day 10 pp testes sections in preleptotene (*a*), leptotene (*b*, *c*), zygotene bouquet (*d*–*g*), and pachytene (*h*) stage. While parental genomes are separated prior to telomere clustering (*a*–*c*), they lie next to each other (*d,* *e*) or overlap in the tightest telomere cluster stage (*f*, *g*). At pachytene, major and minor sat signals partially overlap (*pink color*) and are distributed together with distal telomeres (*green*) over the nuclear periphery. DAPI is false colored and shown in *gray*; nuclear outline is roughly indicated by a *stippled line*. Images represent maximum projections of image stacks. *Bar* 10 μm

One long-standing hypothesis is that formation of the telomere bouquet at the onset of zygotene is instigating homologue pairing (Scherthan 2001). Hence, we investigated the 3D distribution of pericentric genomes prior to and during the bouquet stage of MMU × MSP F1 hybrid spermiogenesis. To follow parental genome redistribution prior to and during the bouquet stage, we enriched for early prophase I substages by obtaining paraffin sections from pubertal MMU × MSP mice 10 and 12 days post partum (dpp), when the rather synchronous onset of male meiosis leads to an accumulation of the short-lived bouquet stage. In agreement, we recovered numerous bouquet stage nuclei among leptotene/zygotene cells of 10 dpp mouse hybrid testes tissue sections (Figs. 2 and 3; S1B, C).

MMU × MSP F1 spermatogonia displayed numerous spatially separate major and minor sat pericentromere signal clusters that were dispersed throughout the nuclear volume, with major/minorsat FISH signals only occasionally touching each other (Fig. 2c). The MMU major sat and the MSP minor sat pericentromere signal clusters were spatially separated up to the onset of zygotene (Fig. 2a,) suggesting pericentric genome separation. MMU pericentromeres formed in average 7 major sat-positive chromocenters, while MSP pericentromeres formed in average 8 minor sat FISH clusters per nucleus (Fig. 2a). PCH clusters that consisted of combined and partially overlapping large major-sat and minor-sat signals were rarely seen (∼1/nucleus) up to the end of leptotene stage. Starting from zygotene stage the number of heterologously paired PCH signal clusters increased to 5–10 minor/major clusters per F1 pachytene nucleus (Fig. 2a, c), reflecting homeologous (homologous in the parents) chromosome pairing. Still there was prominent higher order PCH clustering leading to less than 20 (the haploid number) heterologously colored pericentric sat FISH signals per hybrid pachytene nucleus (Fig. 2a).

**Fig. 4:**
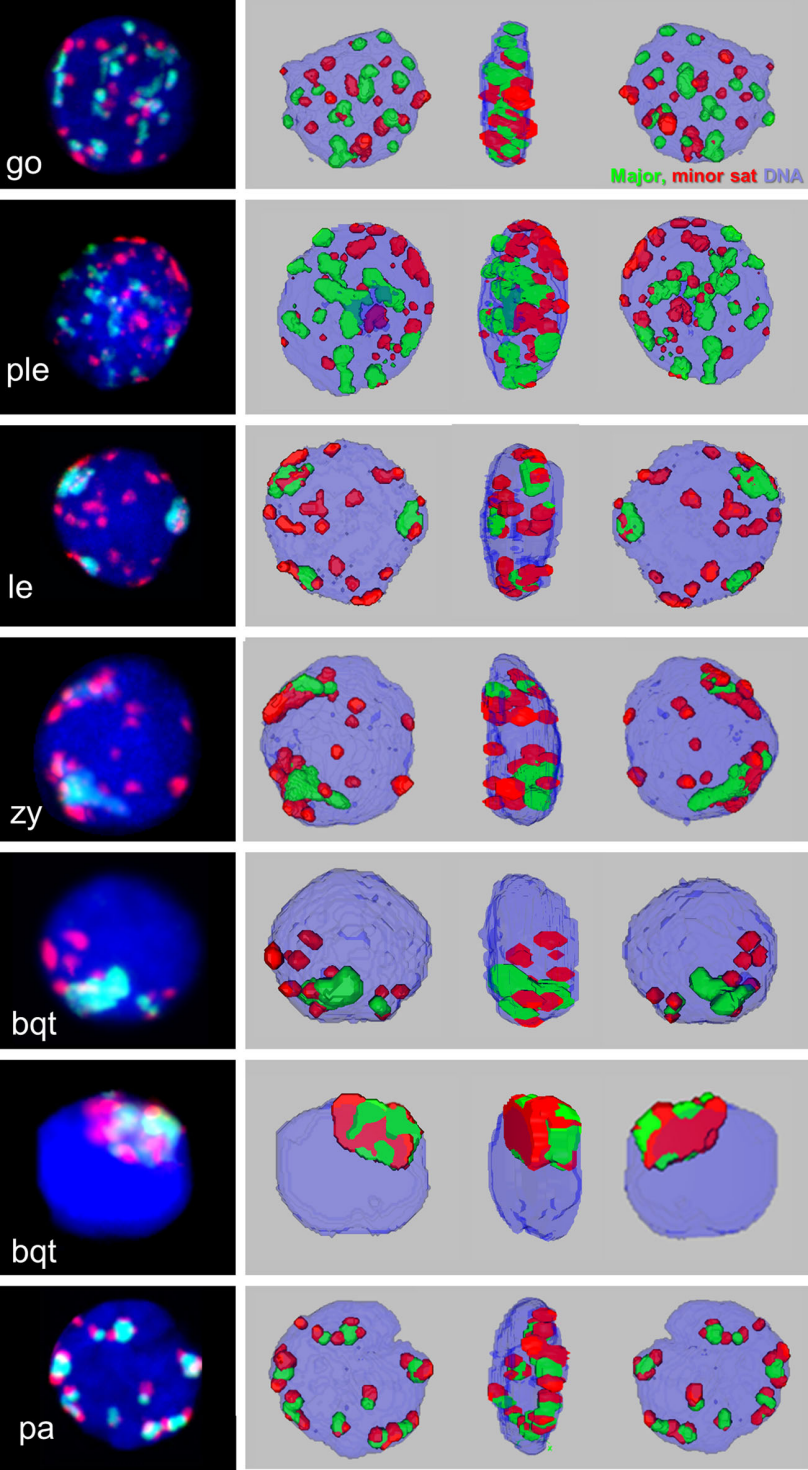
3D reconstructions of MMU × MSP F1 prophase I nuclei from confocal optical sections showing the distribution of minor sat (*red*) and major sat (*green*) signals in early prophase I spermatocytes. Corresponding RGB Z projection images are shown to the left. Spermatogonia (*go*) and preleptotene (*ple*) nuclei show numerous often separate signals, while leptotene (*le*) and zygotene (*zy*) nuclei display reduced numbers of major sat (MMU)clusters; minor sat pericentromeres (MSP)still are dispersed. In the bouquet stage (*bqt*), the previously distant parental pericentric genomes congregate in a limited nuclear space. A tight bouquet stage nucleus (below) displays intensive chromatin intermingling. Homeologous pairing in pachytene (*pa*) nuclei is reflected by numerous *red*/*green* signal doublets as revealed in a pachytene (*pa*) nucleus

### The bouquet congregates spatially separate parental pericentric genomes

Next, we investigated the spatial distribution of the parental genomes from leptotene to the telomere clustering (bouquet formation) at the onset of zygonema more closely. Mouse chromosomes (2*n* = 40) all are acrocentric and centromeres are abutted with a proximal telomere (Kipling et al. 1991). Triple FISH of minor and major sat, and (TTAGGG)_7_ telomere probes in combination with γH2AX immunofluorescence (IF; Fig. 3; S1B) revealed the gradual congregation of telomeres and the associated parental pericentromere domains during bouquet formation (Fig. 3b), leading to 1–3 large MMU major satellite signals in early MMU × MSP F1 bouquet cells, while several minor satellite signals were still distributed in a distinct nuclear sector (Figs. 2c *ii–iv*, 3b and 4). Tight telomere clustering during the bouquet stage concentrated all telomeres and pericentric chromosome regions in a small sector of the zygotene nucleus (Figs. 2c *v* and 3*e–g*), with generally one major and several minor sat FISH signal clusters being in close contact with each other (Figs. 2c *v, vi*, 3*f*, *g* and 4; S1B’*iii*). In pachytene nuclei without marked telomere clustering pericentric FISH signals of both parental genomes formed partially overlapping major and minor sat signal couples (Figs. 2c *vii* and 3*h*, S1B’ *iv*).

3D reconstruction from confocal Z-stacks (Fig. 4) and quantitative image analysis revealed that the overlap of minor and major sat FISH signal pixels was in average 25 % of total sat signal/nucleus in spermatogonia nuclei (Fig. 2b; *n* = 18) expressing the gonia-specific marker PLZF (Fig. 2c *i*) (Buaas et al. 2004; Costoya et al. 2004). In leptotene nuclei, there was in average 34 % major and minor sat FISH signal overlap (*n* = 17; Fig. 2b). It seems possible that some of the occasional faint MMU minor sat signals embedded in major sat PCH may also have contributed to some of the signal overlap observed.

In the tight bouquet stage, minor and major FISH signal pixel overlap peaked at 90 % (*n* = 8; Fig. 2b), indicating that telomere clustering (which in mouse drags PCH behind) is inducing intensive chromatin interactions at chromosome termini (Fig. 2c; Fig. 3*g*; Fig. 4). In post-bouquet zygotene nuclei signal overlap dropped to 54 % (*n* = 19) and 60 % in pachytene nuclei (*n* = 25), reflecting the partial overlap of pericentric genomes due to side-by-side pairing of homeologous chromosomes in these stages (Figs. 2c *vii*, 3*h* and 4). The signal overlap pattern may also be influenced by the more relaxed 3D chromatin organization of zygotene and pachytene chromosomes (Scherthan et al. 1998).

Interestingly, we observed that sat FISH to nuclei prepared from testis suspensions usually revealed a larger number of sat signal clusters per nucleus relative to nuclei from paraffin sections, which is likely owing to the lack of tissue context and flattening of nuclei in testis suspensions. Nonetheless, the observed meiotic redistribution patterns of MMU and MSP pericentric genomes were similarly revealed in both preparation types. It is evident that parental pericentric chromosome sets become separated during pre/leptotene stages and that telomere clustering during the bouquet stage congregates the two spatially separated parental PCH genomes.

### Euchromatic chromosome arms undergo presynaptic pairing during leptotene

**Fig. 5:**
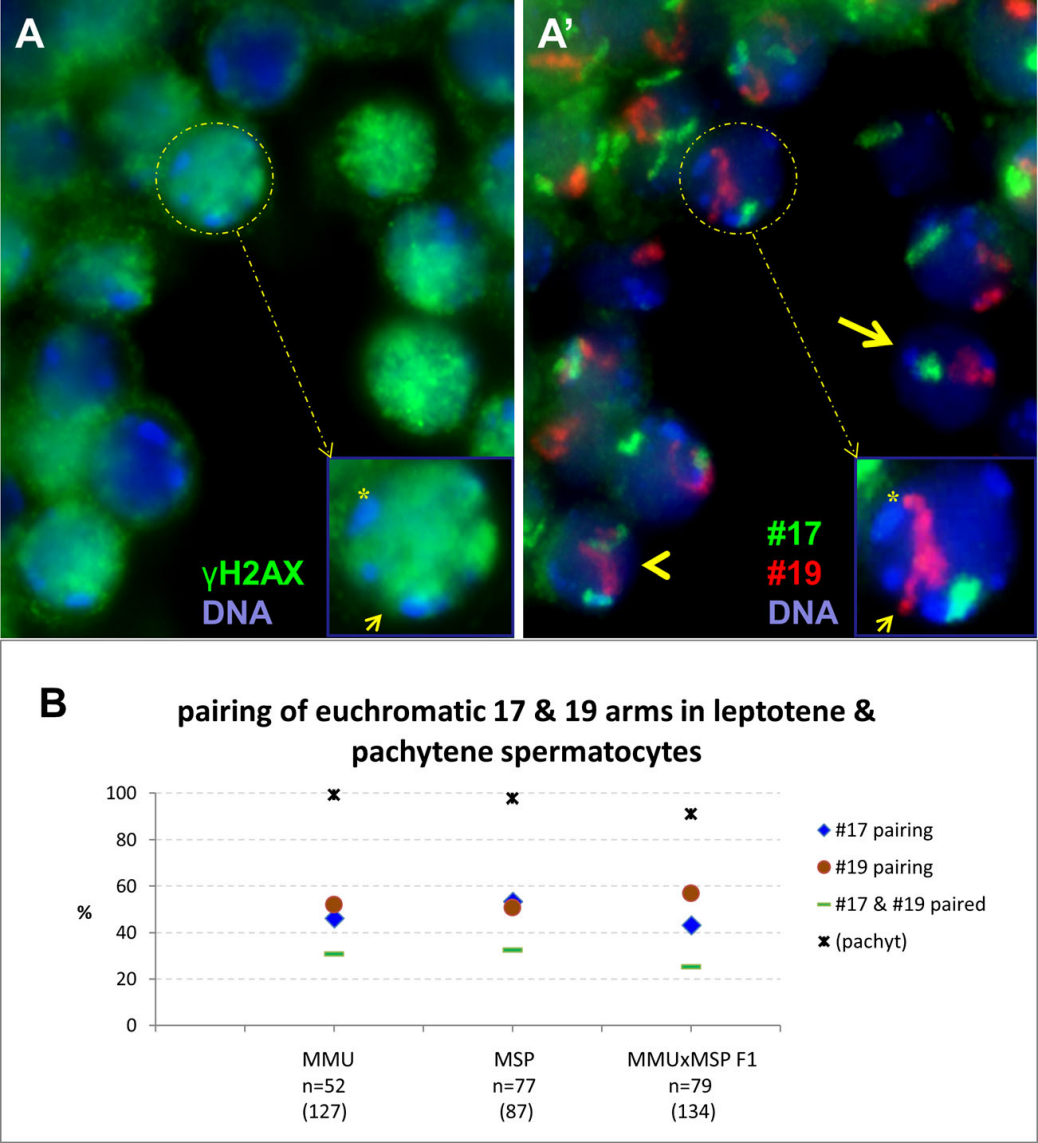
Two-color painting of the euchromatic portions of chromosomes #17 (*green*) and #19 (*red*) in leptotene spermatocytes of 10 dpp MMU × MSP F1 testes paraffin sections. **a** γH2AX IF staining shows the overall γH2AX fluorescence (Cy5, false-coloredin *green*) of leptotene spermatocytes. *a’* Same detail showing the painted euchromatic chromosome arms in the leptotene spermatocytes indicating significant presynaptic pairing of chromosome territories in leptotene spermatocytes. A leptotene nucleus displays pairing of both chromosomes #17 and #19 (*arrow*, one signal/nucleuseach), while others show #17 separated and #19 paired (*arrow head*), or vice versa. The *inset* shows a leptotene nucleus where chromosome #17 FISH signals are paired and the #19 territories separated at their proximal ends, one of which is associated with a DAPI-bright(thus MMU) pericentric heterochromatin cluster (*asterisk*), while the other reaches to the nuclear periphery without a distinct DAPI signal (*small arrow*), and hence, represents the proximal MSP #19. **b** Frequencies (%) of chromosome signal pairing in leptotene spermatocytes of MMU, MSP, and their F1 hybrid. The *green lines* represent the frequency of nuclei with both chromosomes #17 and #19 paired (one signal for each chromosome pair/nucleus). The *red dots* show the total frequency of #19 pairing and the *blue diamond* that of #17 pairing (one signal/nucleus). While the parents show similar high frequency of presynaptic leptotene pairing, the F1 hybrid leptotene spermatocytes display slightly diverged pairing frequencies for the two chromosome territories. The *asterisks* give the level of pairing (both chromosome pairs) in pachytene (pachyt) spermatocytes (*n* indicates the number of leptotene nuclei analysed; numbers of pachytene spermatocytes investigated are in *brackets*). The images shown represent maximum projections of Z stacks

While parental pericentric genome portions were spatially separate in MMU × MSP F1 leptotene cells, they were eventually congregated by telomere clustering in the bouquet stage. There are observations that suggest that euchromatic portions of homologous chromosomes undergo presynaptic pairing prior to bouquet formation in *M. musculus* meiosis (Boateng et al. 2013; Ishiguro et al. 2014). In contrast to the parental PCH genome portions that share sequence homology and undergo considerable higher order clustering (see above), euchromatic parts of MMU chromosomes seem to undergo prealignment in nearly half of leptotene spermatocytes (Ishiguro et al. 2014). This opens the possibility that parental PCH genome distribution in the interspecific F1 hybrids is either not representative for MMU spermatogenesis or that the redistribution of PCH regions during the pairing process is different from the euchromatic portion of the genome. To address the possibility of differential 3D organization of pericentric heterochromatin on the one hand and euchromatic chromosome arm regions on the other hand, we performed a two-color painting of mouse chromosomes #17 and #19 on testis paraffin tissue sections. Chromosome painting probes highlight the euchromatic chromosome portions but spare PCH regions containing repetitive DNAs (Lichter et al. 1988; Pinkel et al. 1988). Chromosomes #17 and #19 were FISH painted in testis tissue sections of parental species and the F1 hybrid. Leptotene nuclei were identified by tissue context and an pan-nuclear γH2AX expression (Fig. 5; Mahadevaiah et al. 2001). Maximum projection images were obtained from image stacks recorded from 14 μm testis tissue sections to minimize signal loss due to sectioning. Chromosomes #19 or #17 were considered paired when there was one paint signal for each specific chromosome (Fig. 5). It was found that the MMU, MSP and MMU × MSP F1 leptotene spermatocytes display presynaptic pairing of the painted euchromatic chromosome arm portions in about 50 % of leptotene nuclei investigated. Both chromosomes were simultaneously paired in the same cell in ∼30 % of leptotene cells in both parental species (Fig. 5b), and a considerable number of cells displayed only one of the two painted chromosome pairs engaged in pairing (Fig. 5). The pairing behavior of #17 and #19 was similar in the spermatocytes of the parental mouse species (Fig. 5b), #19 displayed higher total pairing values in the F1 hyprid relative to #17 (Fig. 5b). The different pairing behavior in the F1 hybrid spermatocytes may be associated with variable heterozygosity and/or different RAD21L cohesin distribution patterns along the homeologous chromosome pairs in the hybrid spermatocytes, which may contribute to homologue recognition and alignment (Ishiguro et al. 2014). Since chromosome mobility during the leptotene/zygotene stage is very dynamic, the chromosome painting data suggest that pairing is asynchronous, dynamic and different from the mode of 3D distribution of pericentromeric heterochromatin. Synaptic pairing, in contrast, involved simultaneous pairing of #17 and #19 homeolog pairs in 99 % and 98 % of *M. musculus* and *M. spretus* pachytene spermatocytes, respectively, while in the MMU × MSP F1 hybrid simultaneous pairing was observed in 95 % of spermatocytes (Fig. 5b) with #17 or #19 chromosomes each being separate in 2.5 % of spermatocytes. In all, it appears that the homotypic unspecific clustering (stickiness) of PCH is distinct from the behavior of the euchromatic portions of meiotic chromosomes. Presynaptic pairing of euchromatic arm portions is likely being mediated by DSB-dependent and independent mechanisms (Boateng et al. 2013; Ishiguro et al. 2014).

### Parent-specific heterochromatin organization may explain preferential clustering of MMU pericentromeres

Higher-order clustering of pericentric heterochromatin leads to chromocenter formation in various mouse cell types including spermatocytes (Hsu et al. 1971; Scherthan et al. 1996). Heterochromatin components have long been hypothesized to contribute to this “stickiness” of PCH (see, e.g. Guenatri et al. 2004 and Hsu et al. 1971). Heterochromatin builds on histone H3 K9 trimethylation (Peters et al. 2001; Tachibana et al. 2007). H3K9me3 associates with heterochromatin protein 1 (HP1) family proteins that are abundant in spermatogenic cells (Tachibana et al. 2007; Yoshioka et al. 2009). Among HP1 proteins, HP1-gamma contributes to PCH clustering in early *M. musculus* spermatocytes (Takada et al. 2011).

**Fig. 6:**
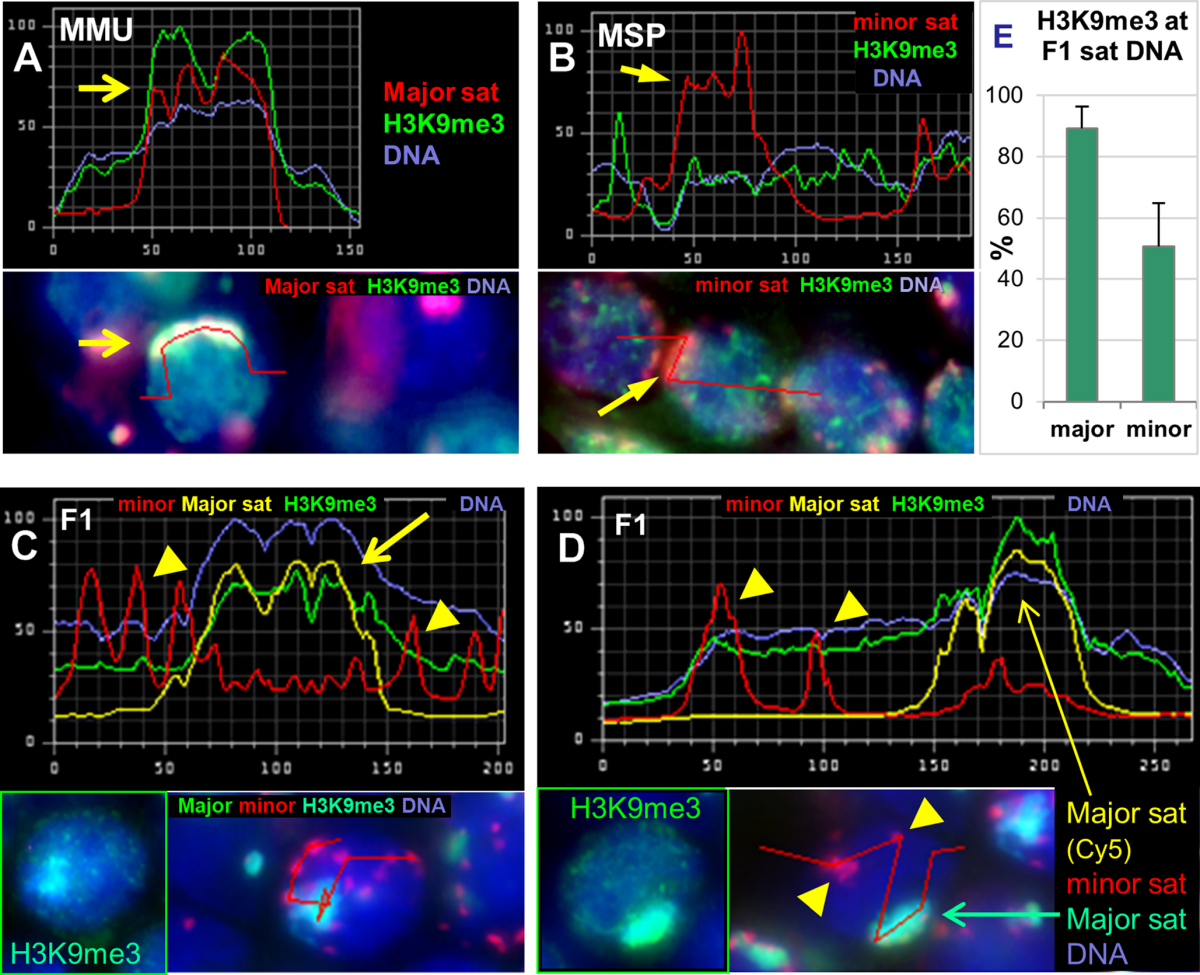
Heterochromatin marks at pericentric heterochromatin (PCH). Fluorescence intensity of H3K9me3 "FITC," marks *(green) *at major sat (Cy5, *yellow*) and minor sat (Cy3, *red*) DNA in **a** MMU, **b** MSP, and **c**, **d** MMU × MSP F1 bouquet spermatocytes of a 10-dpptestis. Fluorescence intensity profile analysis shows an increased abundance of H3K9me3 at major sat signals that colocalize with DAPI-bright(*blue*) major sat clusters (*arrows*; **a**, **c**, **d**), while MSP minor sat signals (*red*; **b**–**d**) show no increase of H3K9me3 fluorescence in the hybrid nucleus (*arrow heads*). The RGB images *below* the graphs show the quadruple stained tissue section nuclei in which the major sat fluorescence is shown in *green* in the image from which the profile (*red line*) was recorded; **c**, **d** the corresponding H3K9me3 IF is shown in the green-framedinset. **e** Relative presence (%) of H3K9me3 fluorescence at FISH labelled major and minor satellite DNA clusters in hybrid spermatocytes. More than 20 nuclei were analysed

Immunofluorescent (IF) staining of H3K9me3 in combination with sat DNA FISH revealed this heterochromatin mark from leptotene up to early pachytene, with a particular enrichment at major satellite-containing DAPI-bright PCH clusters in MMU spermatocytes (Fig. 6a; S2). MSP spermatocytes, on the other hand, failed to show a particular enrichment for H3K9me3 at minor sat DNA clusters (Fig. 6b). The enrichment for H3K9me3 was also present at MMU major sat DNA clusters in F1 hybrid nuclei (Fig. 6c, d), with major sat DNA clusters displaying an approximately 1.8-fold stronger H3K9me3 fluorescence signal intensity compared to minor-sat marked MSP PCH clusters (Fig. 6e; *n* = 14 leptotene spermatocytes). In contrast, di-methyl-H3K9 was relatively evenly distributed throughout early meiotic nuclei (Figs. S2; S3A). Previously, it has been observed that the chromatin modifier methyl CpG-binding protein MeCP2 contributes to pericentromere clustering during terminal differentiation of somatic MMU cells (Brero et al. 2005). In MMU spermatogenesis, we observed by IF to paraffin testis sections that MeCP2 was absent from preleptotene up to pachytene stage parental and hybrid spermatocytes (Fig. S3A, and not shown). A similar observation was made for HP1-alpha loading that commenced at MMU PCH in mid pachytene stage (Fig. S3B).

**Fig. 7:**
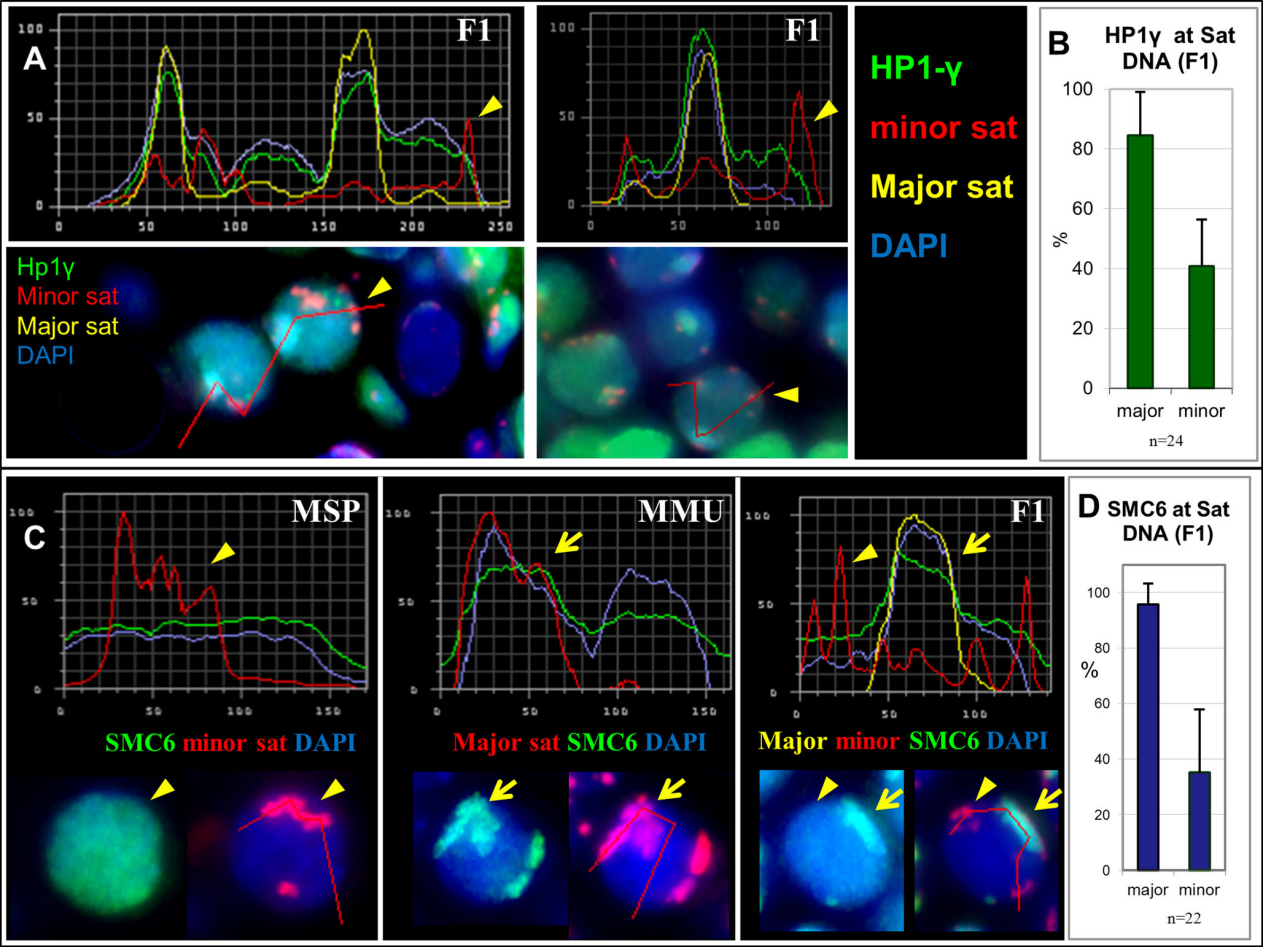
Heterochromatin proteins at pericentric heterochromatin (PCH). Fluorescence intensity of HP1gamma protein (FITC, *green*) at major sat (Cy5, *yellow*) and minor sat (Cy3, *red*) DNA in **a** MMU × MSP F1 hybrid bouquet spermatocytes. **b** Relative presence (%) of HP1gamma fluorescence at FISH-labelledmajor and minor satellite DNA clusters in hybrid spermatocytes. **c** SMC6 association with major sat-containingMMU PCH. **c** Fluorescence intensity profile analysis shows the absence of increased SMC6 fluorescence at minor satPCH (MSP, *red*) of a MSP bouquet spermatocyte. MMU PCH is enriched for SMC6 at major sat PCH (MMU)signals (*arrow*, *red*). F1 hybrid bouquet nucleus (F1)maintains enrichment for SMC6 at MMU major sat PCH, while MSP minor sat signals (*red*, *arrow head*) show no increase of SMC6 fluorescence in the hybrid nuclei (*arrow heads*). The RGB images below the graphs show the tissue section nuclei from which the intensity profile (*red line*) was recorded. **d** Relative presence (%) of SMC6 fluorescence at FISH-labelledMMU major and MSP minor satellite DNA clusters in F1 hybrid spermatocytes

HP1-gamma, on the other hand, was observed to be enriched at major sat-containing MMU PCH of leptotene and zygotene spermatocytes in MMU (not shown) and MMU × MSP F1 hybrids (Fig. 7a).

In all, we found that major satellite DNA of early meiotic MMU pericentromeres accumulates a higher level of H3K9 tri-methylation relative to MSP minor sat DNA. This, together with loading of HP1-gamma (this report, Takada et al. 2011) may explain the stickiness of the MMU pericentric regions as reflected by fewer and larger major sat chromocenters in early spermatocytes of MMU and the F1 hybrid (Figs. 1c and 2a).

### SMC5/6 complex is enriched at major sat DNA

In maize meiosis, it has been observed that early meiotic centromere pairing depends on a structural maintenance of chromosomes 6 (SMC6) protein homologue (Zhang et al. 2013). In this respect, it is of note that *M. musculus* spermatocytes display an enrichment of the mouse SMC5/6 complex at H3K9me3-positive pericentric heterochromatin where it is thought to exclude this repetitive DNA-containing PCH from recombination (Verver et al. 2013). Furthermore, the SMC5/6 protein complex loads to SCs and meiotic centromeres (Gómez et al. 2013). We, therefore, investigated the enrichment of the SMC5/6 complex at minor and major sat DNAs in parental and MMU × MSP F1 bouquet spermatocytes. Like for H3K9me3, we observed a preferential enrichment of SMC6 at major sat-containing pericentromeres of MMU spermatocytes, and at the MMU major sat PCH of F1 hybrid spermatocytes (Fig. 7c, d). In contrast, SMC6 was evenly distributed throughout MSP leptotene and zygotene nuclei, and﻿  the minor sat-positive PCH of the MSP parent failed to show particular enrichment for SMC6 in early F1 spermatocytes (Fig. 7c, d). It is thus intriguing to speculate that the association of the SMC5/6 complex together with increased H3K9me3 at MMU major sat DNA also may contribute to the preferential stickiness of major sat-carrying MMU pericentric heterochromatin.

## Discussion

The role of centromere coupling and telomere clustering for the meiotic homologue pairing process has been difficult to tackle. In plants, especially in those with large genomes, centromere coupling, together with subsequent tight telomere clustering (bouquet formation), seems to facilitate the meiotic pairing process at the synapsis level (Martinez-Perez et al. 1999; Richards et al. 2012; Zhang et al. 2013). It has been assumed that centromere coupling may play a role in holding off pericentric regions from homology search and recombination initiation (Obeso and Dawson 2010) and in the removal of inappropriate chromosome interactions prior to homology search (Klutstein and Cooper 2014). Centromere clustering may have taken the role of the telomere bouquet in species (*Drosophila*) that do not form a telomere bouquet (Takeo et al. 2011). The coupling/clustering of pericentromeric regions early in meiosis may have developed because pericentromeres are available as large chromatin hubs of general sequence and protein homology before a defined and specific meiotic chromosome architecture with protein cores has been installed. In mice with acrocentric karyotypes, the physical neighborhood of pericentromeric heterochromatin to telomere sequences (Kipling et al. 1991) may favor PCH clustering because telomere motility from leptotene onwards will lead to collisions of adjacent pericentromeres inducing clustering by PCH stickiness.

In mammalian spermatocytes with a more heteromorphic karyotype, differential clustering of pericentric regions of metacentric vs acrocentric chromosomes has been noted. This is the case for the domestic pig where the spatial separation of PCH regions of metacentric and acrocentric chromosomes has supposedly supported the development of distinct pericentric satellite DNA families associated with the two different chromosome morphs (Jantsch et al. 1990). Premeiotic PCH clustering may generate spatial proximity according to chromosome size and morphology, leading to presorting of particular chromosomes which in turn may support the presynaptic alignment of homologs in mouse leptotene nuclei (Ishiguro et al. 2014). In support, HP1γ (encoded by *Cbx3*) and G9a methyltransferase mutant spermatogeneses show defects for early meiotic PCH clustering, homologue pairing and synapsis (Takada et al. 2011).

In MMU × MSP F1 interspecific hybrid spermatogenesis, we noted that parental pericentric heterochromatin surprisingly undergoes a spatial separation prior to the telomere clustering in the bouquet stage. This is reminiscent of the sorting of early meiotic chromosomes into non-homologous centromere couples in yeast meiosis (Obeso and Dawson 2010; Tsubouchi and Roeder 2005). In plants, centromere interactions have been observed in somatic cells (Martinez-Perez et al. 1999), the leptotene stage of maize (Zhang et al. 2013) and at the leptotene/zygotene transition in *Brachypodium distachyon* (a monocot) meiosis (Wen et al. 2012).

**Fig. 8:**
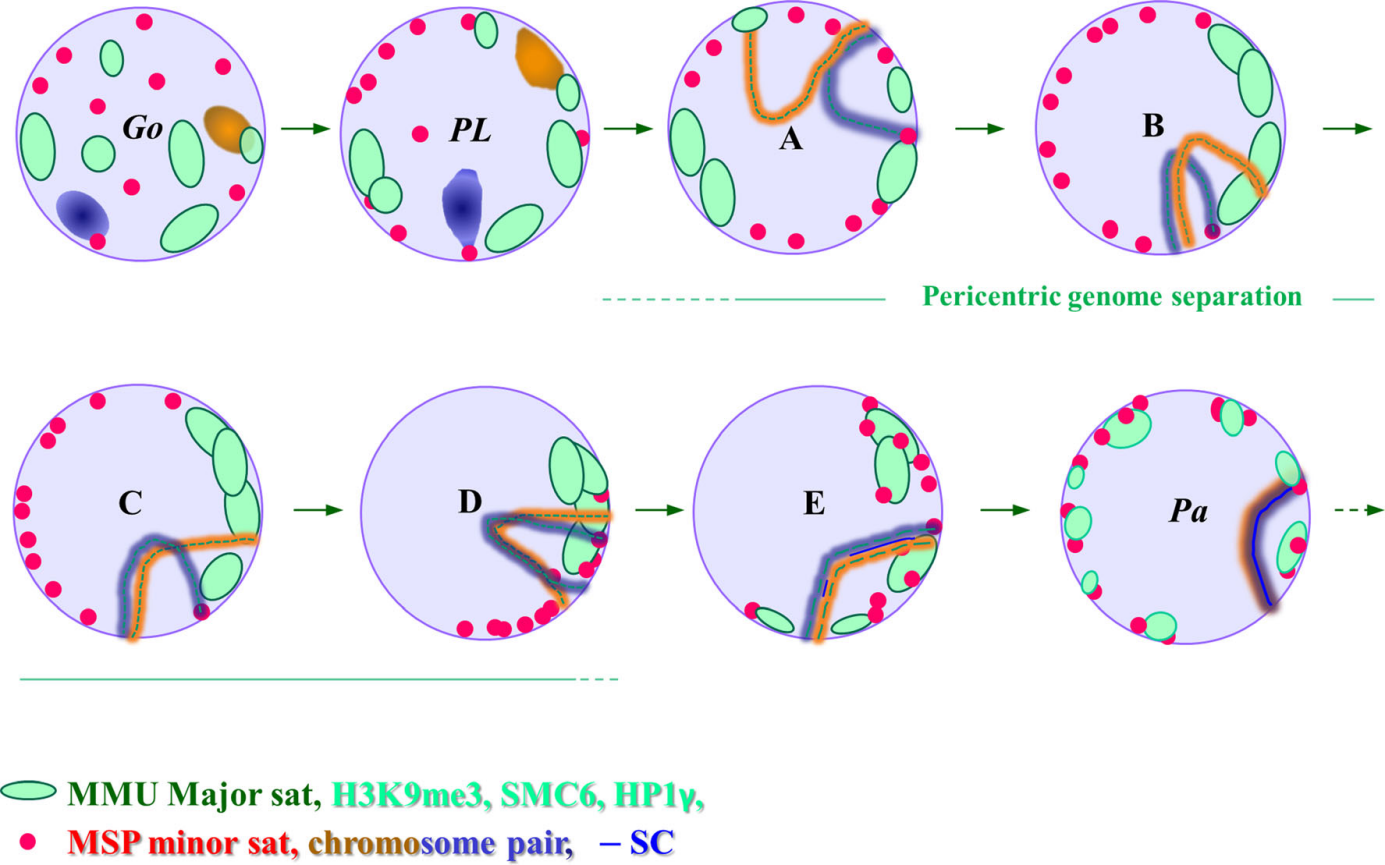
Cartoon of the course of parental genome redistribution during MMU × MSP F1 male prophase I (c.f. Figs. 2c and 4). *Go* In spermatogonia, homologous chromosomes (homeologs in the hybrid) form compact territories that are usually spatially separated (*blue* and *brownish ovals* represent a typical homeologue pair). *PL* During preleptotene, pericentromeres (MMU major sat, *green*; MSP minor sat, *red*) and telomeres (not shown for simplicity) transit to the nuclear envelope to which telomeres attach, while chromosome territories remodel and start to elongate (see, Scherthan et al. 1998). *A*–*C* In leptotene nuclei chromosome territories elongate, assemble cores and can undergo presynaptic pairing at euchromatic arm portions, while MMU major sat PCH forms few large clusters and the minor sat-taggedMSP pericentromeres are typically distributed in nuclear sectors void of major sat signals indicating separation of PCH of the parental genomes. Note that in mouse meiosis all prophase PCH is peripheral due to NE attachment of the adjoining telomeres. MMU major satellite PCH is particularly enriched for H3K9me3, HP1γ, and SMC5/6 complex proteins (*light green*) and undergoes more intimate clustering in pre-bouquet stages (*A*–*C*), while MSP minor sat PCH is present in numerous clusters. Tight telomere clustering during zygotene (*D*) merges the previously separated parental PCH clusters (c.f. Fig. 3e–g) and pairs still separated hom(e)ologous chromosome portions or chromosomes. The *large letters* denote the typical PCH distribution patterns observed, whose frequency is given in Table 1. In tight bouquet nuclei, we often noted a side-by-sidearrangement of parental PCH signals (pattern *D*) prior to their intimate interaction (c.f. Figs. 2c and 4). Entry (*A*) and exit (*E*) patterns were less frequent among the 60 early prophase cells studied (Table 1), likely owing to the brevity of these stages. Pachytene nucleus (*Pa*) showing typical PCH distribution with smaller major and minor sat-containing PCH clusters and one bivalent due to synaptic pairing. The dynamics of the PCH redistribution during prophase I can be viewed in an animated gif movie https://ws.molgen.mpg.de/ws/103735/PCH_genome_separation_hscherthan.gif

The genome separation observed here in pre-bouquet spermatocytes of a mammalian hybrid adds a new act to the choreography of meiotic homology pairing, which seems to generate a favorable genome topology in the early meiotic nucleus facilitating homology search (Fig. 8, Table 1). In rodents, parental pericentromere clustering is dependent on heterochromatin functions as mice defective for histone methylation and HP1-γ deposition show defective meiotic chromosome behavior (Peters et al. 2001; Takada et al. 2011). For specific homologue/homeologue recognition, DSB-dependent and independent mechanisms seem to be at work in the euchromatic chromosome portions (arms), as Spo11 mutant mice display disturbed synapsis (Baudat et al. 2000; Romanienko and Camerini-Otero 2000) but still undergo significant homologue prealignment (Boateng et al. 2013). Recent data in the mouse suggest that specific meiotic cohesin patterns mediate DSB-independent presynaptic homologue recognition and alignment (Ishiguro et al. 2011, 2014). The latter mechanism may also be at work in our hybrid mice where it appears to be restricted to the euchromatic parts of meiotic chromosomes, since the euchromatic portions of chromosomes 17 and 19 underwent presynaptic pairing in about 50 % of leptotene spermatocytes of *M. musclus*, *M. spretus* and in their F1 hybrid, which contrasts with concurrent parental pericentromere clustering and separation. The parental presynaptic PCH separation may draw on a particular stickiness of the major satellite-rich MMU PCH, as the MMU major sat cluster numbers were well below those of the minor sat-tagged MSP PCH in the parents and hybrid leptotene and zygotene spermatocytes. The particular MMU PCH stickiness during pre-bouquet stages may be mediated by the relative enrichment of MMU heterochromatin for H3K9me3 marks and the ensuing HP1-gamma accumulation. Accordingly, spermatocytes deficient for HP1γ or histone methyltransferases show defects in PCH clustering (Peters et al. 2001; Tachibana et al. 2007; Takada et al. 2011). Interestingly, DAPI-bright heterochromatin clusters are completely dissolved when methylation of H3K9 is impossible (Pinheiro et al. 2012).

**Table 1:** PCH distribution patterns and frequency

PCH distribution patterns in early F1 spermatocyte nuclei						
Tubule no.	A	B	C	D	E	Spermatocytes studied
1	3	6	5	4	2	20
2	3	3	2	4	2	14
3	1	2	3	3	2	11
4	2	6	2	3	2	15
%	15	28.33	20	23.33	13.33	

Quantification of parental PCH congregation in the bouquet stage. The columns A-E correspond to the parental PCH distribution patterns as depicted in Fig. 8. The frequency of the observed minor/major sat distribution patterns (Fig. 2c) was derived from four 10 dpp testis tubules (2 mice). The gradual congregation culminates in mixing of the parental PCH and initiation of synaptic pairing in the tight bouquet stage (see text)

Moreover, we observed a particular enrichment of the structural maintenance of chromosomes 6 (SMC6) protein at major sat-positive PCH in MMU and the F1 hybrid. The SMC5/6 protein complex is involved in the regulation chromatin structure, DNA repair (De Piccoli et al. 2009) and has been implied in the exclusion of HR repair at PCH (Chiolo et al. 2011; Verver et al. 2013). Smc5/6 complex loading seems to be independent of meiotic cohesin and may have a role in SC structure (Gomez et al. 2013). Of interest, a SMC6 homologue has been found to contribute to early centromere pairing in maize meiosis (Zhang et al. 2013). Altogether, the preferential enrichment for heterochromatin components and Smc5/6 complex proteins at MMU major sat DNA may contribute to the particular stickiness of the MMU pericentromeres that drives higher order clustering and self-aggregation leading to PCH genome separation in early meiotic prophase of hybrid mice.

Pericentric genome separation was eventually disbanded by telomere clustering during the bouquet stage, which forced pericentric genomes (due to their physical association with motile meiotic telomeres) into a limited nuclear sector (Fig. 8). Since euchromatic parts of the genome undergo significant pairing in pre-bouquet MMU spermatocytes (Boateng et al. 2013), likely during leptotene (this investigation; Ishiguro et al. 2014), it appears that the telomere bouquet likely serves as a matchmaker for homologs or portions thereof that fail to align during leptotene and for heterochromatic peri-centromeres that obviously are spared from the cohesin-dependent presynaptic homologue pairing process. In agreement, the PCH regions of human metacentric chromosomes 3 are particularly compacted during leptotene (Scherthan et al. 1998) and the subtelomeric but not pericentric regions are required for correct pairing of large plant chromosomes (Corredor et al. 2007). In all, alignment of chromosome cores and their agglomeration at the bouquet base seems to facilitate synapsis initiation between previously distant homologous chromosomes. The importance of telomere clustering for mammalian homologue pairing is underlined by the observation that disruption of telomere/nuclear envelope attachment or uncoupling of telomeres from the force-generating cytoskeleton both leads to defective homologue pairing/synapsis and spermatocyte death (Ding et al. 2007; Horn et al. 2013), while presynaptic homologue recognition remains intact (Ishiguro et al. 2014). In all, centromere coupling, preclusion of pericentric heterochromatin from homology search and telomere clustering seem to be collectively at work for an efficient chromosome pairing process in meiosis.

## Materials and methods

### Mice

F1 hybrid males were obtained from an interspecific cross between female *M. musculus* (MMU) strain B6C3F1 laboratory mice, and male European wild mice. *Mus spretus* (MSP) SEG/Pas mice were a kind gift of Xavier Montagutelli, Institute Pasteur, Paris, France. Mice were kept at the MPI for Molecular Genetics mouse facility in compliance with local animal welfare laws, guidelines and policies. Animal handling was performed according to approved guidelines.

### Fluorescence in situ hybridization

We used FISH with differentially labelled pericentromeric satellite DNAs to distinguish between maternal and paternal pericentric heterochromatin in MMU × MSP hybrid spermatogenic cells in paraffin sections. A Cy5-conjugated major satellite 42 bp oligomer TTT TCC ACC TTT TTC AGT TTT CCT CGC CAT ATT TCA CGT CCT-3′ tagging MMU pericentromeres (Scherthan and Cremer 1994) and a Cy3-labelled minor satellite 42mer: AAG GTG TAT ATC ATA GAG TTA CAA TGA GAA ACA TGG AAA ATG-3′ tagging MSP pericentromeres (Scherthan et al. 2000) were obtained from Invitrogen. Telomeres were detected with (TTAGGG)_7_ Fluorescein-labelled oligomeres as described (Scherthan and Cremer 1994). The satellite probes detected the respective parental pericentromeres in a MMU × MSP hybrid cell line established from the F1 animals (Figure S1).

For FISH to paraffin sections, preparations were dewaxed in xylene, treated with 100 mg/ml RNase A (Sigma) in 2× SSC at 37 °C for 15 min, followed by heating in 50 mM Citrate buffer for 1 h at 94 °C. After cooling down, slides were briefly air dried and 1.5 μl of hybridization mixture (1× SSC, 2 ng/μl minor and major sat oligos) was applied to each section on the slide and sealed under a 10 mm Ø coverslip with rubber cement. To reveal predominantly large satellite clusters, slides were hybridized in a moist chamber for 3–4 h at 37 °C without further denaturation. Thereafter, slides were washed three times for 5 min in BT buffer (0.15 mM NaHCO_3_, 0.5 % Tween 20) at RT. DNA was counterstained with DAPI in the mounting medium. For further details see (Scherthan and Cremer 1994).

#### Immunostaining and FISH

Immunofluorescent staining was combined with FISH as described (Scherthan et al. 1996) using citrate buffer-pretreated paraffin sections (Ahmed et al. 2012). Sections were first dewaxed and treated for 1 h in 94 °C 50 mM Na citrate, and after cooling down, washed and hybridized with satellite oligonucleotides for 4 h, washed again in PBS and immunostained using primary and secondary antibodies as indicated in Table 2.

#### Chromosome painting

For chromosome painting of paraffin section nuclei, probes for MMU chromosome 17 and 19 were obtained commercially (MetaSystems) and used in combination with Cy5-labelledγH2AX IF to detect the frequency of homolog pairing during leptotene. Sections were dewaxed as described above. Probes in hybridization solution were sealed under a cover slip on the sections, denatured for 3 min at 80 °C on a hot plate, followed by hybridization for >2 days at 37 °C in a moist chamber. Finally, slides were washed 4× 3 min in 0.05 x SSC, 0.25 % Tween20 at 40 °C and embedded in antifade containing DAPI (Vectashield).

**Table 2:** Antibodies

Antibodies	Supplier	Order no.	Dilution
Primary antibodies			
Mouse anti-γ-H2AX	Millipore	JBW301	1/250
Rabbit anti-MeCP2	Millipore	07-013	1/200
Goat anti-HP1-alpha	abcam	ab77256	1/200
Rabbit anti-HP1-beta	Genetex	gtx106418	1/100
Mouse anti-HP1-gamma	Euromedex	2MOD-1G6-AS	1/500
Mouse anti-H3K9me2	abcam	ab1220	1/200
Rabbit anti-H3K9me3	abcam	ab8898	1/400
Rabbit anti-SMC6L1	abcam	ab18039	1/200
Secondary antibodies			
Goat anti-mouse Alexa-488	Mobitec	A11017	1/500
Donkey anti-goat-FITC	Dianova	705-225-147	1/400
Donkey anti-rabbit-Cy3Fab	Dianova	711-167-003	1/1,000

#### Microscopy

Images of IF-stained cells were recorded using the ISIS fluorescence image analysis system (MetaSystems, Altlussheim). In some cases, 3D image stacks (step size 0.4 μm) were converted to maximum projection images. Fluorescence profile analysis was done using the measurement option in the ISIS imaging software (MetaSystems).

Confocal laser scanning imaging and collection of focus stacks was performed on a Leica TCS SP5 confocal laser-scanning microscope (kindly provided by H. Leonhard, L-M-U Munich, Germany) equipped with Plan Apo 63x/1.4 NA oil immersion objective (voxel size 50 × 50 × 200 nm), and lasers with the excitation lines 405, 488, 561, 594 and 633 nm. Quantitative image analyses was done in 3D reconstructions from confocal image stacks using ImageJ (http://imagej.nih.gov/ij/) or Nemo (Iannuccelli et al. 2010) as recommended by the developer (https://www-lgc.toulouse.inra.fr/nemo/).

## Electronic supplementary material

Below is the link to the electronic supplementary material.

Fig. S1 (PDF 737 kb)

Fig. S2 (PDF 66 kb)

Fig. S3 (PDF 287 kb)

ESM 1 (GIF 1523 kb)
