# Supplementary material for: Contrasting behavior of heterochromatic and euchromatic chromosome portions and pericentric genome separation in pre-bouquet spermatocytes of hybrid mice
Source: Chromosoma. 2014 Aug 15;123(6):609–24. doi: 10.1007/s00412-014-0479-4 (PMC4226931; doi:10.1007/s00412-014-0479-4)
Supplement: Supplementary file 1 — (PDF 737 kb) [file 412_2014_479_MOESM1_ESM.pdf]

Supplementary Figures

Fig. S1

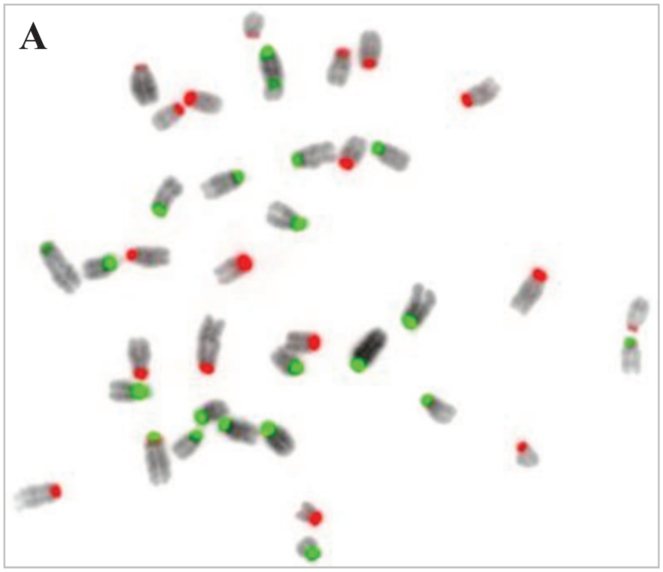

Fig. S1A: Partial metaphase of a transformed MMUxMSP F1 cell line after differential FISH tagging of major (green) and minor (red) satellite DNA. *M. spretus* chromosomes are labeled red at their pericentromeres, while *M. musculus* chromosomes show strong green pericentromeric major sat FISH labeling. DNA was counterstained with DAPI which is shown in gray.

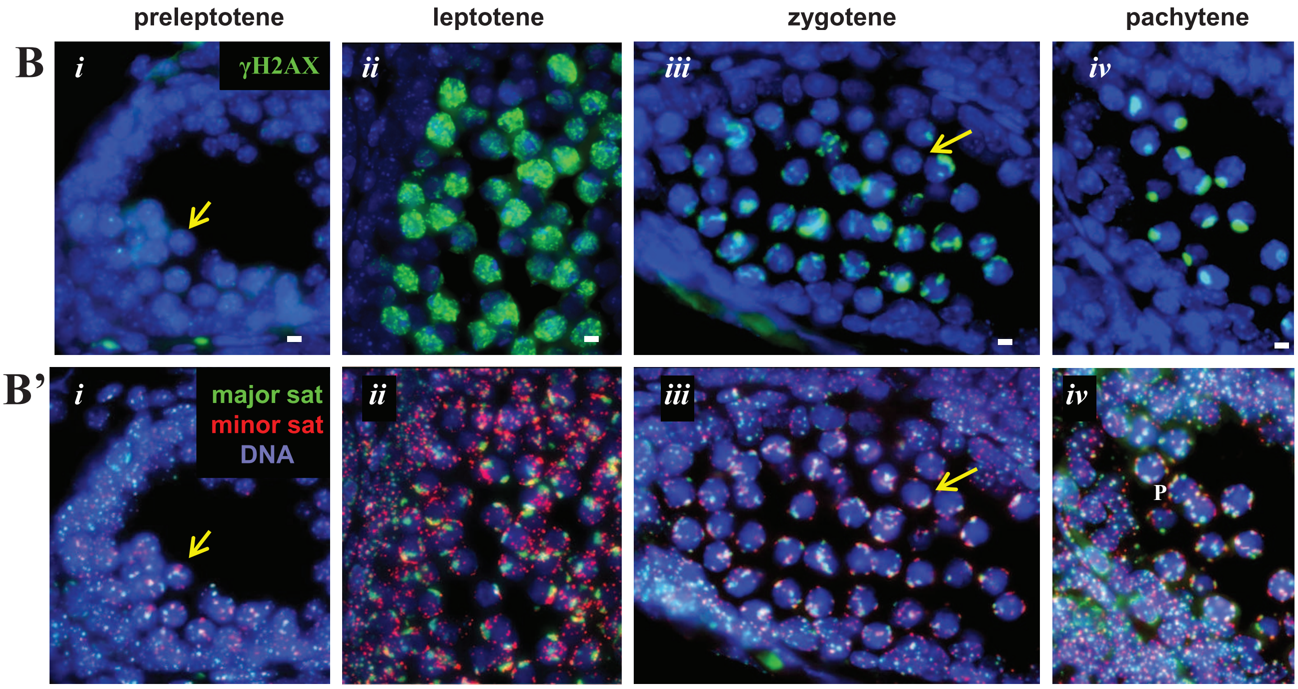

Fig. S1B,C: Parental pericentromere genome distribution as revealed by minor (red) and major (green) sat DNA FISH and stage-specific γH2AX staining (see, Mahadevaiah et al., 2001) to paraffin testis sections. (B) Images of testes tubules with different stages of early prophase I substages according to the γH2AX IF patterns in paraffin sections, these images correspond to the FISH images shown in row C. Bars: 5μm. (B*i*) γH2AX IF shows the onset of DSB formation which coincides with (B'*i*) distinct clustering of pericentromeres seen as bright red (minor sat, MSP) and green (major sat, MMU) FISH signal clusters at the pre/leptotene transition (representative nucleus arrowed). (B,B'*ii*) Leptotene nuclei displaying strong γH2AX fluorescence exhibit few large green major and numerous red minor sat signal clusters. (B,B'*iii*) Major and minor sat clustering during bouquet formation (arrow) in zygotene nuclei as indicated by a patchy γH2AX IF pattern. (B,B'*iv*) Pachytene nuclei (P) exhibiting a γH2AX-positive XY body and several peripheral heterologous FISH signal clusters indicating synaptic chromosome pairing (cf. Fig.1).
