# Supplementary material for: Contrasting behavior of heterochromatic and euchromatic chromosome portions and pericentric genome separation in pre-bouquet spermatocytes of hybrid mice
Source: Chromosoma. 2014 Aug 15;123(6):609–24. doi: 10.1007/s00412-014-0479-4 (PMC4226931; doi:10.1007/s00412-014-0479-4)
Supplement: Supplementary file 2 — (PDF 66 kb) [file 412_2014_479_MOESM2_ESM.pdf]

Fig. S2

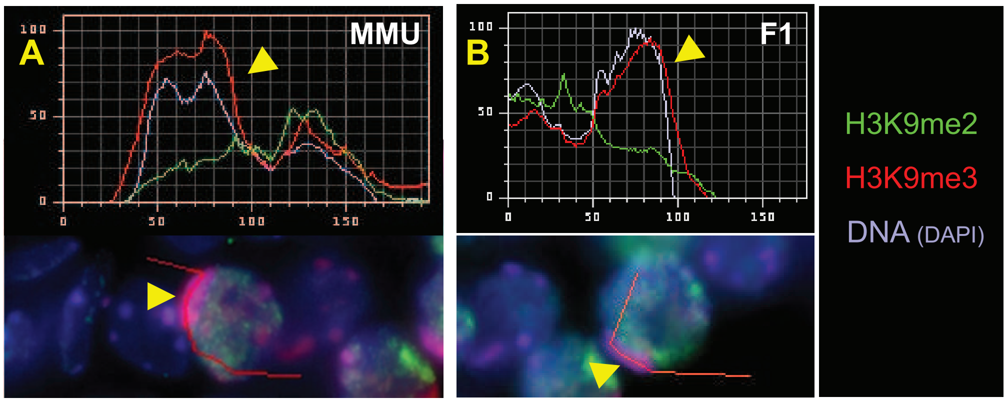

Fig.S2: Di- and Tri-methylation of histone H3K9 in MMU and MMUxMSP F1 bouquet spermatocytes. (**A**) H3K9me3 (Cy3, red) and H3K9me2 (Cy2, green) fluorescence intensity at PCH clusters of bouquet spermatocyte nuclei of a 6 month-old MMU testis, and (**B**) a MMUxMSP 10d F1 testis paraffin section. Fluorescence intensity profiles with the same color code show that H3K9me3 is particularly enriched at DAPI-bright (blue) MMU PCH (arrow head), while H3K9me2 follows a more uniform nuclear distribution in early spermatocytes.
