# Supplementary material for: Contrasting behavior of heterochromatic and euchromatic chromosome portions and pericentric genome separation in pre-bouquet spermatocytes of hybrid mice
Source: Chromosoma. 2014 Aug 15;123(6):609–24. doi: 10.1007/s00412-014-0479-4 (PMC4226931; doi:10.1007/s00412-014-0479-4)
Supplement: Supplementary file 3 — (PDF 287 kb) [file 412_2014_479_MOESM3_ESM.pdf]

Fig. S3

A

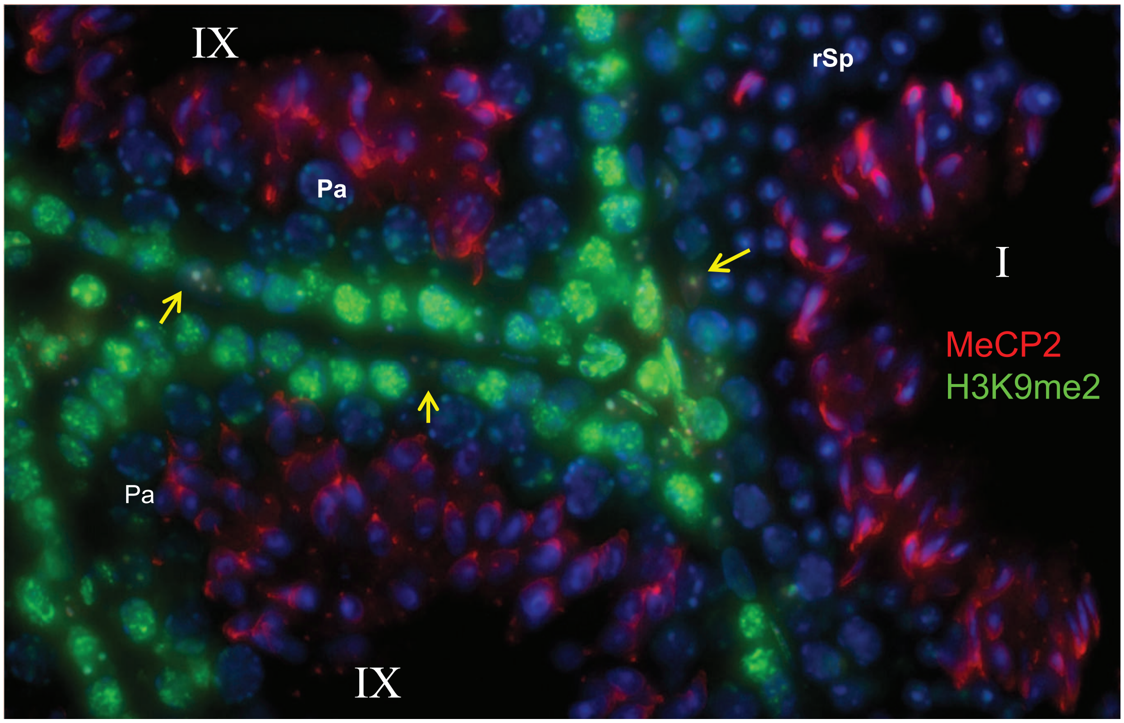

Fig. S3A: MeCP2 (red) and H3K9me2 (green) heterochromatin marks in a testis section of a MMU 35dpp mouse. Both heterochromatin marks are seen at the PCH clusters of Sertoli cells (arrows) and Leydig cells. MeCP2 is absent from prophase I nuclei. H3K9me2 strongly labels leptotene/zygotene nuclei and is largely restricted to PCH clusters of pachytene spermatocytes (Pa; stage IX tubules) and to PCH of round spermatids (rSp) in a stage I tubule.

B

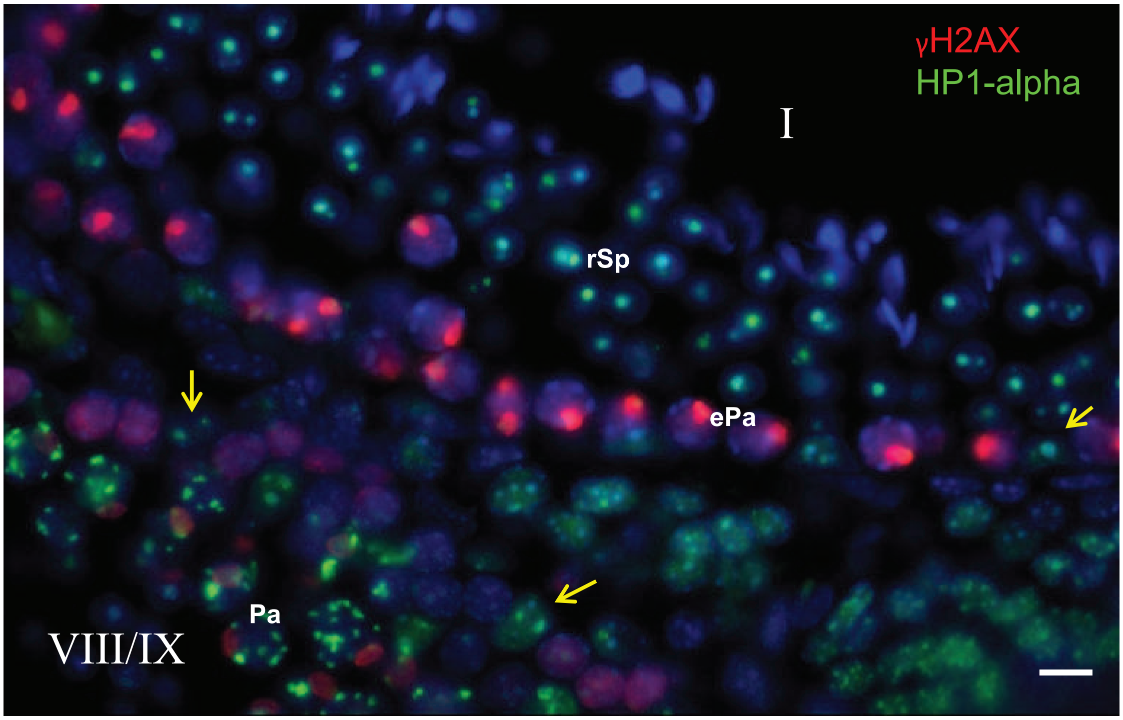

Fig. S3B: HP1-alpha (green) and γH2AX (red) in a testis section of a MMU 35dpp mouse. Hp1α stains PCH clusters of Sertoli cells (arrows) and first appears at PCH clusters of mid-pachytene spermatocytes (Pa). HP1α strongly locates to PCH clusters of round spermatids (rSp). ePa, early pachytene. Bar: 10μm.
