# Supplementary figures and images for: Contrasting behavior of heterochromatic and euchromatic chromosome portions and pericentric genome separation in pre-bouquet spermatocytes of hybrid mice
Source: Chromosoma. 2014 Aug 15;123(6):609–24. doi: 10.1007/s00412-014-0479-4 (PMC4226931; doi:10.1007/s00412-014-0479-4)

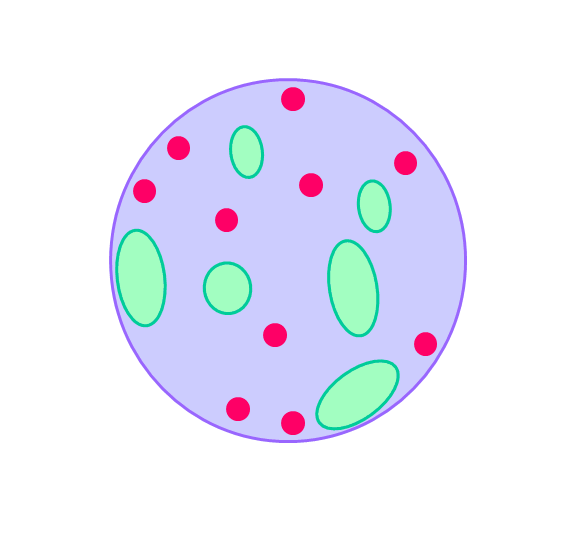

Supplement: Supplementary file 4 — (GIF 1523 kb) [file 412_2014_479_Fig9_ESM.gif]
